# Time-restricted feeding prevents memory impairments induced by obesogenic diet consumption, via hippocampal thyroid hormone signaling

**DOI:** 10.1016/j.molmet.2024.102061

**Published:** 2024-11-06

**Authors:** Jean-Christophe Helbling, Rachel Ginieis, Pierre Mortessagne, Mariano Ruiz-Gayo, Ioannis Bakoyiannis, Eva-Gunnel Ducourneau, Dominique Ciocca, Illona-Marie Bouleté, Alexandre Favereaux, Aurélia Ces, Enrica Montalban, Lucile Capuron, Freddy Jeanneteau, Guillaume Ferreira, Etienne Challet, Marie-Pierre Moisan

**Affiliations:** 1 Univ. Bordeaux, INRAE, Bordeaux INP, NutriNeurO, UMR 1286, Teams NutriPsy & FoodCircus, Bordeaux, France; 2 Department of Health and Pharmaceutical Sciences, Facultad de Farmacia, Universidad San Pablo-CEU, CEU Universities, Madrid, Spain; 3 Chronobiotron, Centre National de la Recherche Scientifique (CNRS), University of Strasbourg, France; 4 Univ. Bordeaux, CNRS, Interdisciplinary Institute for Neuroscience, IINS, UMR 5297, Bordeaux, France; 5 Institute of Cellular and Integrative Neurosciences, CNRS, University of Strasbourg, France; 6 Institut de Génomique Fonctionnelle, Université de Montpellier, INSERM, CNRS, Montpellier, France

**Keywords:** Obesity, Cognition, Chrono-nutrition, T3, Circadian rhythms, Gene expression

## Abstract

**Objective:**

The early consumption of calorie-rich diet disrupts circadian rhythms and has adverse effects on memory, yet the effects of time-restricted feeding (TRF) and the underlying molecular mechanisms are unknown. Here, we set out to identify the behavioral and molecular circadian rhythms disruptions generated by juvenile obesogenic diet consumption and their restoration by TRF in male mice.

**Methods:**

Metabolic rhythms were measured by indirect calorimetry and memory performances by behavioral tasks. Hippocampal translatome (pS6_TRAP), enrichment and co-regulated gene network analyses were conducted to identify the molecular pathways involved in memory impairments and their restoration by TRF. Differential exon usage analyses, mass spectrometry and pharmacological intervention were used to confirm thyroid hormone signaling involvement.

**Results:**

We show that four weeks of TRF restore the rhythmicity of metabolic parameters and prevents memory impairments in mice fed a high fat-high sucrose (HFS) diet since weaning, independently of body fat levels. Hippocampal translatome and differential exon usage analyses indicate that impaired memory of mice under *ad libitum* HFS diet is accompanied by reduced thyroid hormone signaling and altered expression of astrocytic genes regulating glutamate neurotransmission. TRF restored the diurnal expression variation of part of these genes and intra-hippocampal infusion of T3, the active form of thyroid hormone, rescues memory performances and astrocytic gene expression of *ad libitum* HFS diet-fed mice.

**Conclusions:**

Thus, thyroid hormones contribute to the TRF positive effects on both metabolism and memory in mice fed an obesogenic diet, highlighting this nutritional approach as a powerful tool in addressing obesity brain comorbidities and paving the way for further mechanistic studies on hippocampal thyroid signaling.

## Highlights

• Time-restricted feeding restores food intake amplitude, mean respiratory exchange ratio and mean energy expenditure but has no effect on locomotor activity.
• Time-restricted feeding on early obesogenic diet consumption restores short-term object location memory and long-term object recognition memory.
• Time-restricted feeding restores part of the hippocampal translatome altered by early obesogenic diet consumption.
• Thyroid hormone signaling in hippocampus contributes to the impaired memory of mice fed obesogenic diet since weaning and its restoration by time-restricted feeding.
• Mice fed obesogenic diet since weaning display altered astrocytic gene expression involved in glutamatergic reuptake that is restored by time-restricted feeding.

## Graphical abstract

**Figure:**
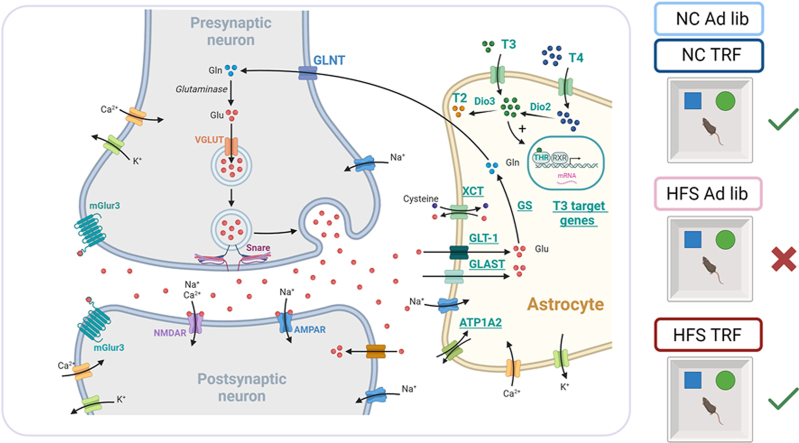

## 1 Introduction

The prevalence of pediatric obesity has plateaued in most high-income countries but remains high, and it continues to rise in low and middle-income countries. Child and adolescent obesity are associated with multiple immediate and long-term negative health outcomes, impacting quality of life and increasing economic costs [1]. The consumption of an obesogenic diet, rich in saturated fat and refined sugar (HFS), at any time of day and night is one of the major causes of obesity, especially among adolescents [2]. HFS diet has deleterious consequences not only on cardiometabolic health but also on brain health and cognitive functions as shown by epidemiological and brain morphological analyses [3,4]. Over the last 15 years, accumulated data in rodent models report the negative impact of such diet consumption on cognitive processes [5,6]. Early life consumption of HFS diet is particularly harmful for the brain that is still under development [7]. The hippocampus has been very much studied for its vulnerability to calorie-rich diet and its consequences on learning and memory. Our group has found that HFS diet impaired hippocampal-dependent memories when started in juveniles, but did not affect memory when started at adulthood [8], through aberrant activity of the hippocampus [9,10]. However, the molecular underpinnings of the HFS diet-induced memory impairments remain unknown.

HFS diet is also known to disturb circadian rhythms at the behavioral and molecular levels, favoring metabolic disturbances [[11], [12], [13], [14]]. In particular, unlimited access to HFS food leads to an increased food intake during the resting phase and a reprogramming of the circadian expression of the transcriptome and metabolome in mice liver [15], suprachiasmatic nucleus of the hypothalamus and in prefrontal cortex [16]. The impact of juvenile HFS diet consumption on molecular circadian rhythms within hippocampus is still unexplored.

Time-restricted eating (TRE) in humans and time-restricted feeding (TRF) in animals, during which time of access to food is restricted to hours of the active phase, without calorie restriction, has emerged as an alternative strategy to protect against obesity and dysmetabolism. Human studies of TRE showed weight and fat loss as well as improved metabolic parameters in overweight and obese adult patients [17,18]. In animal models, re-alignment of food intake onto circadian rhythms by TRF restored the oscillations of liver transcripts related to metabolism and improved metabolic health [[19], [20], [21]]. In models of Alzheimer disease, TRF was also beneficial, restoring part of the hippocampus circadian transcriptome and improving memory [22]. The beneficial effect of TRF on juvenile HFS diet-induced long-term memory alteration and hippocampal circadian transcriptome remained to be evaluated.

To fill these gaps, we identified behavioral and hippocampal molecular circadian disruptions in our model of juvenile consumption of HFS diet and the beneficial effect of TRF.

## 2 Materials and methods

### 2.1 Animals and time-restricted feeding (TRF)

Male C57BL/6J mice aged 3 weeks (Janvier Labs, France) were randomly divided into groups of 6 per cage (45 × 25 × 20 cm, containing a cardboard house, nesting material and a small wooden stick) and had *ad libitum* access to a normal chow diet (NC; 2.9 kcal/g; 8% lipids, 19% proteins, 73% carbohydrates; A04, NEUTRAL) or a high-fat and sugar diet (HFS; 4.7 kcal/g; 45% lipids, 20% proteins, 35% carbohydrates of which 50% is sucrose; D12451, Research Diet). All animals were housed in a temperature-controlled room (22 ± 1 °C) maintained under a 12 h light/dark cycle (lights on at 7:30 am; Zeitgeber time (zt) 0) and had free access to food and water for 8 weeks. From week 8 to week 12, NC and HFS mice were divided in 4 groups (NC ad lib and HFS ad lib groups with unlimited food access; NC TRF and HFS TRF with time-restricted access to food from zt11 to zt1. Animal weighing was performed once per week for most cohorts, fat mass (in grams) was measured by nuclear magnetic resonance (NMR, minispec LF90 II, Bruker, Wissembourg, 67166) after 8 and 12 weeks of diet exposure, i.e. before and after the TRF treatment, in one cohort of mice. Behavioral procedures started after 12 weeks of diet exposure. All animal care and experimental procedures were in accordance with the INRAE Quality Reference System and French (Directive 87/148, Ministère de l’Agriculture et de la Pêche) and European legislations (Directive 86/609/EEC). They followed ethical protocols approved by the Region Aquitaine Veterinary Services (Direction Départementale de la Protection des Animaux, approval ID: B33-063-920) and by the local animal ethic committee of Bordeaux CEEA50 (APAFIS #22684 and #32843). Every effort was made to minimize pain and discomfort and reduce the number of animals used.

### 2.2 Calorimetry and feeding patterns

Daily patterns of energy expenditure and respiratory exchange ratio were determined in individual metabolic cages using an open-circuit indirect calorimetry system (Addenfi, Les Cordeliers, Paris, France). Energy expenditure and respiratory exchange ratio, defined as the ratio of produced CO_2_ production and consumed O_2_, were calculated using AlabSuite (v1.55, Addenfi). This set-up also recorded feeding behavior using an automated weighing system of the feeder (detection threshold of 0.05 g), as well as total locomotor activity estimated by weighing sensors integrated into the bottom of the cage. Mice were acclimatized to the metabolic cages during 2 consecutive days. Measurements and analyses were performed during the third day.

### 2.3 Behavioral procedures

Behavioral activity of each session was recorded through a camera connected to a monitor outside the experimental room allowing the experimenter to visualize the mice during the session and to evaluate and analyze the behaviors later on. Animals were handled every day (1–2 min) for 3 days before the beginning of each behavioral procedure.

#### 2.3.1 Object recognition memory (ORM)

This test was performed as described [23] in order to evaluate long-term novel object memory. For the training session, mice were placed into an open field arena (40 cm × 40 cm) containing two identical objects (cylinders) and were left to explore these called familiar objects until they reached a criterion of 20s of total exploration for both objects. Exploration time was registered when the snout of the mouse was directed towards the objects from a distance shorter than 2 cm, and climbing on the objects was not recorded as exploration. During the test session, performed either 24 h (or 48 h for T3 infusion experiment) after the training session, mice were placed in the same arena and exposed to two objects, one familiar (cylinder) and one novel object (lab glass bottle). The time exploring these objects was again quantified until a criterion of 20s of total exploration for both objects was reached. Thereafter, preference for the novel object was calculated as the percentage of time exploring the novel object over total time exploring both objects. A maximum cutoff of 10 min was established for both sessions, and animals that explored the objects less than 20s were excluded from the analysis. Exploration time was manually quantified, by a trained experimenter outside the experimental room.

#### 2.3.2 Object location memory (OLM)

This test was performed in order to evaluate object location memory. For the training session, mice were placed into an open field arena (40 cm × 40 cm) containing two identical objects (cylinders) and were left to explore these called familiar objects until they reached a criterion of 20s of total exploration for both objects. Exploration time was registered when the snout of the mouse was directed towards the objects from a distance shorter than 2 cm, and climbing on the objects was not recorded as exploration. During the test session, performed 1 h after the training session, mice were placed in the same arena and exposed to the same objects (cylinders) with one of them moved to the opposite angle of the arena. The time exploring these objects was again quantified until a criterion of 20s of total exploration for both objects was reached. Thereafter, the preference for the displaced object was calculated as the percentage of time exploring the displaced object over the total time exploring both objects. A maximum cutoff of 10 min was established for both sessions, and animals that explored the objects less than 20s were excluded from the analysis. Exploration time was manually quantified, by a trained experimenter outside the experimental room.

#### 2.3.3 Contextual fear conditioning (CFC)

An unpaired fear conditioning protocol was used as an alternate Pavlovian conditioning procedure capable of robustly producing contextual fear memory in mice as described in [24]. Briefly, 100 s after being placed in the conditioning context, animals received a shock (0.3 mA, 1 s), then, after a 20 s interval, a tone (70 dB, 1000 Hz, 15 s) was delivered; finally, after a 30 s delay, the same tone then the same shock spaced by a 30 s interval were presented. After 20 s, animals were returned to their home cage. The next day, after a 24 h delay, mice were submitted to two memory retention tests (Day 2). In the tone re-exposure test, mice were placed in the neutral familiar context during 6 min (min) divided in three successive sessions: one before (first 2 min), one during (next 2 min), and one after (2 last min) tone presentation. Two hours later, mice were submitted to the contextual re-exposure test: they were placed for 6 min in the conditioning context without any tone or foot-shock.

#### 2.3.4 Elevated plus maze (EPM)

The elevated plus-maze apparatus consisted of two opposing open arms (30 cm × 8 cm) and two opposing closed arms (30 cm × 8 cm × 15 cm) extending from a 8 cm × 8 cm central platform and elevated 1 m from the ground. Each mouse was first placed onto the central platform facing an open arm and then left to freely explore the apparatus for 5 min. The number of entries in the open or closed arms, as well as the time spent on the various sections of the maze, was recorded. The percentage of time spent in the open arms ((time in the open arms)/(time in the open + closed arms) × 100) was used as an index of anxiety, whereas total number of entries in the closed arms was used as an index of locomotor activity.

### 2.4 Thyroid hormone concentrations within mouse hippocampus

TH concentrations were measured in Dr A Boelen’s lab (Amsterdam, The Netherlands), from frozen whole hippocampus or from plasma by UPLC-MS/MS as previously described [25]. Detection limits were 3 nmol/L for T4 and 0.1 nmol/L for T3.

### 2.5 T3 intra-hippocampal infusion

After 10 weeks under NC (*n* = 15) or HFS (*n* = 20), mice were anesthetized with air/isoflurane (4.5% induction; 1.5% maintenance) at 1L/min, injected with the analgesic buprenorphine (Buprecare, 0.1 mg/kg s.c. in 0.9% saline) and the non-steroidal anti-inflammatory drug carproxifen (Carprofene, 20 mg/kg s.c. in 0.9% saline) and were placed into the stereotaxic apparatus (David Kopf Instruments). The scalp was shaved, disinfected with 10% povidone iodine and locally anaesthetized with a subcutaneous injection of lidocaine (Lurocaine 20 mg/ml, 0.1 ml non-diluted) and a midline incision made over the top of the skull. Bregma and lambda were located and marked to determine implant position. Bilateral implant (8 mm stainless steel guide cannula) coordinates were 2 mm posterior posterior and 1 mm ventral to Bregma and 1.3 mm distal from the midline on both sides targeting dorsal CA1. Guide cannulae were secured in place with dental cement. Mice were kept on a heating pad until recovery. Mice were single housed for 2 days and their body weight and behavior were closely monitored during the 4 days following the procedure. Then, they were housed in groups of 2 mice per cage and behavioral tests started 10 days after surgery, enabling optimal recovery. A volume of 0.3 μl of T3 (3,3′,5-Triiodo-L-thyronin, Sigma-T2877) or its vehicle was infused (flow rate: 0.1 μl/min), as a single infusion, bilaterally in the dorsal CA1 (4 ng/μl per side dissolved in 0.9% saline solution), 15min before starting the ORM training session, using silicone tubing connected to a peristaltic pump (PHD22/2000 Syringe Pump Infusion, Harvard Apparatus, Massachusetts, USA) and protruding 1 mm below cannulae tip. The dose of T3 and the timing of infusion were based on Sui et al.’s study [26]. After behavior, the placement of cannulae in dorsal CA1 region of the hippocampus was verified histologically. The memory performance was tested 48 h after the training session of the ORM task. For gene expression, mice were culled 1 h after the training session of the ORM task, i.e. 1 h 25 min after T3 infusion (T3 infusion + 15 min, ORM training 10 min +1 h) (see Figure 6G).

### 2.6 Hippocampi collection

Mice were culled by decapitation and hippocampus was dissected out on ice [27] then placed in LoBind tube (Eppendorf, EP0030108078-250 EA). Tissues were then snap-frozen by submersion in liquid nitrogen prior to storage in a −80 °C freezer.

### 2.7 Translating ribosome affinity purification (TRAP) mRNA isolation

For pS6 RNA-sequencing (pS6-TRAP-Seq) experiments, hippocampi were removed from −80 °C freezer on ice to prevent any thawing and immediately complemented with 1 ml of cold homogenization buffer (10 mM HEPES [pH7.4], 150 mM KCl, 10 mM MgCl_2_, 2 mM DTT, 0.1 complete Protease Inhibitor Cocktail (Sigma, 11836170001), 100U/ml RNasin Ribonuclease Inhibitors (Promega, N2515), 100 μg/ml Cycloheximide). Tissues were disrupted with TissueLyser (Qiagen) for 15s–30 Hz. Homogenates were transferred in a new 1.5 ml tubes and centrifuged for 10 min at 1000 g at 4 °C. The supernatants were transferred to a new tube and NP40 10% (90 μl) was added and incubated on ice for 5 min with gentle mixing. Samples were centrifuged for 10min at 10000 g at 4 °C. 350 μl of RLT buffer (Qiagen, 74034) was added to 50 μl of supernatants (as input samples), homogenates vortexed for 20–30s and snap-frozen in liquid nitrogen prior to storage in −80 °C freezer until RNA purification. RNA from Input samples, reflecting hippocampus whole transcriptome, were purified and used to confirm pS6-TRAP enrichment by qPCR (data not shown). 500 μl of supernatant was incubated with 1:25 anti-pS6 244–247 (ThermoFisher, 44-923G) (20 μl) and incubated for 1.5 h at 4 °C with constant rotation. During anti-pS6 incubation, affinity purification beads were prepared where 200 μl of Protein G Dynabeads (Thermofisher, 10009D) were washed 3 times for 10min in 200 μl of Beads Washing Buffer (10 mM HEPES [pH7.4], 300 mM KCl, 10 mM MgCl_2_, 1% NP40) and finally incubated for 10min in 200 μl of Supplemented Homogenization Buffer (homogenization buffer supplemented with 1% NP40). Using magnet, beads were collected and homogenate/anti-pS6 solutions were added to beads for 1 h incubation at 4 °C with constant rotation. Following incubation, RNA bound to beads were washed 4 times for 10min at 4 °C in 200 μl of Wash Buffer (10 mM HEPES [pH7.4], 350 mM KCl, 10 mM MgCl_2_, 2% NP40, 2 mM DTT, 100U/ml RNasin Ribonuclease Inhibitors, 100 μg/ml Cycloheximide), during the final wash, beads were placed onto the magnet and moved to room temperature. After the final wash 350 μl of RLT buffer was immediately added to beads. Sample tubes were removed from the magnet and vortexed vigorously for 30s and incubated for 10min at room temperature. After removing beads using the magnet, mRNA was purified using RNeasy PLUS Micro kit (Qiagen, 74034). RNA integrity was assessed with 1 μl of each mRNA-purified sample using RNA 6000 Pico kit (Agilent, 5067-1513) on an Agilent Bionalyzer with RNA Integrity Number (RIN) score up to 9 and mRNA quantity was measured using Nanodrop spectrophotometer. An average of 150 ng of RNA per sample was obtained.

### 2.8 RNA sequencing and data analysis

Pooled equimolar library was synthesized at the Transcriptome facility using 50 ng of TRAP-mRNA using Illumina Stranded mRNA Prep (Illumina, 20040534) and IDT for Illumina RNA UD Indexes Set B and Set C (Illumina, 20040554 and 20040555). Profiling libraries were assessed with LabChip GX Touch HT Nucleic Acid Analyzer (PerkinElmer, Revvity), with HT DNA NGS 3K Reagent Kit (PerkinElmer, CLS960013) onto DNA X-Mark Chip (PerkinElmer, CLS144006). Then libraries were quantified by qPCR onto LightCycler 480 (Roche) with NEB Next Library Quant Kit for Illumina (NEB, E7630L) and KAPA Library Quantification Kit (Roche, KK4854). Finally, libraries were sequenced at the PGTB facility (https://doi.org/10.15454/1.5572396583599417E12) with NextSeq 2000 P3 reagents (100 cycles) (Illumina, 20040559) onto NextSeq 2000 sequencer platform. Paired-end sequencing reads of 50bp resulted in an average of 60 million paired-end reads. Raw reads were processed using trimgalore (v0.6.7), cutadapt (v3.4), mapped against *Mus musculus* GRCm39 using HISAT2 aligner (v2.2.0) and quality checked with MultiQC (v3.9.5) through workflow Nextflow (v21.10.6) and nf-core/rnaseq (v3.6) pipeline onto genotoul bioinformatics platform Toulouse (Bioinfo Genotoul, https://doi.org/10.15454/1.5572369328961167E12). For differential analysis, reads mapped to unique transcripts were counted with featureCounts (Subread v2.0.3) annotation module. With Rstudio (Rversion 4.2.2), raw counts were filtered to keep genes with at least 10 counts across 73 samples. Differentially expressed genes (DEG) were determined by the R Bioconductor package DESeq2 (v1.38.1) [28] using negative binomial distribution with a model grouping all 73 samples. For Weighted Correlation Network Analysis (WGCNA, v1.72-1) [29], ORM+1 h (*n* = 6) and HC-ZT3 (*n* = 6) groups raw counts for each diet were analyzed separately. For each diet, filtered reads were normalized using DEseq2 variance stabilizing transformation (vst) function and were filtered to keep only genes with a variance larger than 0.025. This resulted in 2147 genes, 2391 genes and 1476 genes respectively for NC ad lib, HFS ad lib and HFS TRF which were used to create modules-associated genes networks. Within each module per diet, module membership and the associated *p-*value was calculated. For differential exon usage (DEU) analysis, reads mapped to unique exon were counted with featureCounts, DEXseq R Bioconductor package (v1.44.0) [30] was used to measure DEU as a surrogate to infer differential splicing events in RNA-Seq data.

RNAseq data and experimental details are available in NCBI’s Gene Expression Omnibus and are accessible through GEO Series accession number GSE267751 (https://www.ncbi.nlm.nih.gov/geo/query/acc.cgi?acc=GSE267751).

### 2.9 Quantification of TRAP-mRNA by real time PCR

qPCR were performed as described [27]. Briefly, 20 ng of RNA from pS6-TRAP experiments were reverse transcribed using SuperScript IV Vilo (Thermo Fisher Scientific), 0.2 ng of cDNA were used for qPCR in 10 μl reaction with appropriate forward and reverse primers specifically designed to target genes of interest (Supplementary Table S7).

### 2.10 Statistical analysis

#### 2.10.1 For metabolic data, behavioral analyses and qPCR data

Data were analyzed in GraphPad Prism 10 (GraphPad Software, Inc., San Diego, CA) unless indicated otherwise. Figure error bars represent the standard error of the mean (SEM). For comparisons, t-tests were used to determine significant differences between means of two group values (eg NC ad lib vs. HFS ad lib) and when more than 2 groups by 2-way ANOVA with Diet and ORM as factors, (or diet and TRF) followed by Tukey post-hoc tests for multiple comparisons, or 3-way ANOVA when 3 factors such as diet, TRF and ORM. For the kinetics of body weight across time a mixed model was used under 3-way ANOVA because of missing values in the table; repeated values were used for weeks of diet exposure. To analyze daily patterns of energy metabolism and feeding, data were fitted to cosinor regressions (SigmaPlot v13, Systat software Inc., San Jose, CA, USA) as follows: [y = A+(B·cos(2·π· (x−C)/24))]where A is the mean level, B the amplitude, C the acrophase of the 24-h rhythm. Significance is indicated as follows: ∗*p* ≤ 0.05, ∗∗*p* < 0.01, ∗∗∗*p* < 0.001, ∗∗∗∗*p* < 0.0001.

#### 2.10.2 For RNA-seq data analysis

Default parameters were used for all bioinformatic tools unless specified. Differential gene expression from RNA-Seq data was determined by DEseq2 using the negative binomial distribution (Wald test) and genes were considered as significantly differentially expressed when the Benjamini-Hochberg adjusted *p*-values <0.05 (=FDR: False Discovery rate). In DEXseq analysis, exons were considered significantly differentially used when adjusted *p*-values <0.05. Functional enrichment analysis such as KEGG pathways and Gene Ontology terms were performed using the R Bioconductor package clusterProfiler (v4.6.2) using enrichKEGG and enrichGO functions. The function compareCluster was used to compare enriched functional categories of gene module. Relaxed significance threshold of adjusted *p*-value <0.2 was used for discovery and pathway analyses. Cell-type enrichment analyses to get specific gene signatures in different brain cell types (neurons, endothelial cell, astrocytes, microglia, oligodendrocyte precursor cells, newly formed oligodendrocytes, and myelinating oligodendrocytes) was performed using RNA-sequencing database [31] and Fisher exact test was used to test enrichment of cell type signatures in genes whin modules provided by WGCNA analysis in each diet. Resulting *p*-values were corrected for multiple tests using the Benjamini-Hochberg method and the significant threshold was set to *p* < 0.05 [32].

## 3 Results

### 3.1 Juvenile ad lib HFS diet induces, and TRF prevents, alteration of circadian metabolic parameters

We compared four groups of male C57Bl/6J mice that were under either a normal chow (NC) or HFS diet (45% kcal fat, 17% sucrose) for 12 weeks since weaning, provided *ad libitum* (ad lib) for the entire 12 weeks (NC ad lib, HFS ad lib) or ad lib for the first 8 weeks followed by time-restricted feeding (TRF) for the last 4 weeks (NC TRF, HFS TRF). We found that TRF significantly reduced the weight gain of the mice under HFS diet at week 11 (*p* = 0.0133) and week 12 (*p* = 0.0003) but did not have an impact on the weight of NC-fed mice (Figure 1A). Accordingly, in HFS mice, TRF was found to significantly reduce fat mass gain over 4 weeks (week 12-week 8), 7.82% vs. 12.04% respectively, for HFS TRF and HFS ad lib, *p* = 0.0001 (Figure 1B). The fat mass gain did not differ between NC TRF and NC ad lib groups, *p* = 0.37 (Figure 1B).

**Figure 1:**
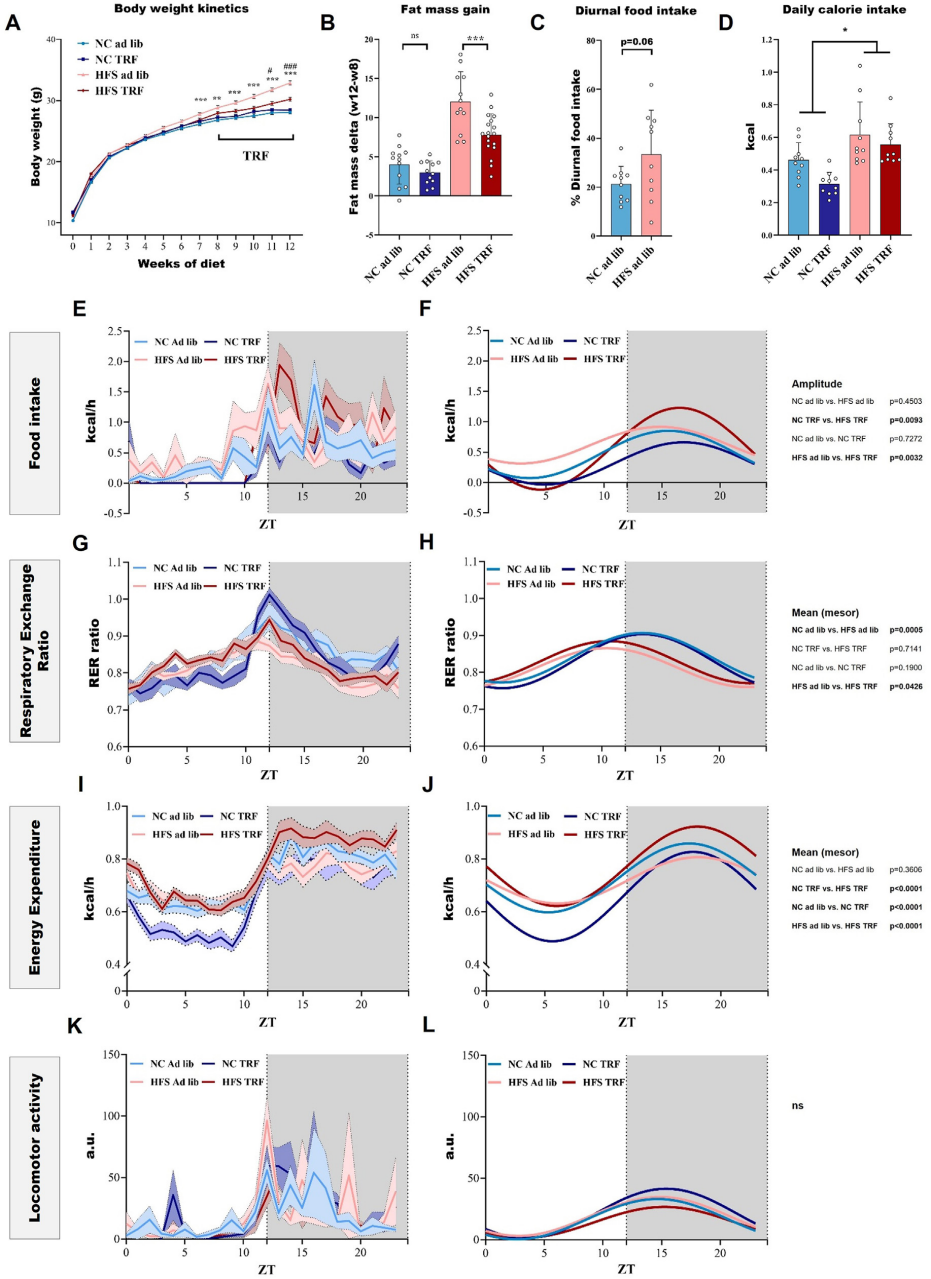
**Juvenile ad lib HFS diet induces, and TRF prevents, alteration of metabolic rhythms. A**: Kinetics of body weight across the 12 weeks of exposure; data from all the mice used in the study were compiled, *n* = 44–92 per group (some cohorts not weighted every week). Data were analyzed by 3-way ANOVA (Diet, TRF and Time (weeks of diet) factors, repeated measure for Time). **B**: Fat mass gain between week 8 and week 12 (measured by EchoMRI), unpaired t-test; *n* = 12 per group except HFS TRF *n* = 18. **E–L**: Metabolic rhythms measured by indirect calorimetry: raw data on the left and cosinor regression on the right. Hourly mean (mesor), amplitude and acrophase data were compared by 2-way ANOVA analyses (Diet and TRF factors), *n* = 10 per group. Data are represented as mean ± SEM. When Diet × TRF interaction is significant posthoc analyses are shown.

Cosinor regressions were used to calculate mesor (hourly mean levels), amplitude (difference between highest and lowest values), and acrophase (peak value) for each metabolic parameter measured by indirect calorimetry. Detailed data and statistics for each metabolic parameter are presented in Supplementary material. From the food intake measurements, we were able to calculate the diurnal percentage of food intake which was 33 ± 5% in HFS ad lib mice versus 21 ± 2% in NC ad lib mice (*p* = 0.06) (Figure 1C). A high variability is observed for diurnal food intake in the HFS ad lib mice which may be due to photophobia or deep sleep during the light phase for the mice exhibiting low diurnal food intake. Total 24 h calorie intake was significantly impacted by HFS but not by TRF, (2-way ANOVA, main diet effect *p* = 0.0017) (Figure 1D). By resynchronizing food intake to day/night cycles, TRF restored food intake amplitude (HFS TRF vs. HFS ad lib, *p* = 0.032, Figure 1E–F), mean RER (HFS TRF vs. HFS ad lib, *p* = 0.043, Figure 1G–H) and mean EE (HFS TRF vs. HFS ad lib, *p* < 0.001, Figure 1I–J). However, TRF had no effect on locomotor activity rhythms (Figure 1K and L).

### 3.2 Juvenile ad lib HFS diet induces memory deficits that are prevented by TRF

Since TRF re-aligned metabolic rhythms onto circadian rhythms in mice under HFS diet, we examined whether the hippocampal-dependent memory impairments previously observed in HFS ad lib mice [9,33] were prevented by TRF. Indeed, TRF prevented the impairments on short-term object location memory (OLM) tested early afternoon (one-sample t test against 50%: *p* < 0.001 for all groups except HFS ad lib; 2-way ANOVA interaction Diet x TRF *p* < 0.0001, HFS ad lib *p* < 0.0001 compared to all other groups, Figure 2A). Previous work showed that long-term (24 h) but not short-term (3 h) object recognition memory (ORM) was altered in HFS mice [10]. TRF prevented alterations on long-term object recognition memory (ORM) in HFS mice, tested in the morning, i.e. light phase (one-sample t test against 50%: *p* < 0.05 for all groups except HFS ad lib and NC TRF; 2-way ANOVA: interaction *p* = 0.0019, HFS ad lib *p* = 0.004 compared to all other groups except NC TRF (Figure 2B). TRF also prevented impairments of ORM in HFS mice when tested in the evening (dark phase) (one-sample t test against 50%: *p* < 0.05 for all groups except HFS ad lib, 2-way ANOVA, main diet effect *p* = 0.033, HFS ad lib *p* < 0.03 compared to all other group except *p* = 0.06 compare to HFS TRF), (Figure 2C). Regarding contextual fear conditioning, another hippocampal-dependent memory test, the impairments observed in HFS ad lib mice, in the morning, were not rescued by TRF (2-way ANOVA, main diet effect, *p* = 0.0018), (Figure 2D). Notably, anxiety-related behaviors were unchanged in the four experimental groups when tested in the elevated plus maze since there was no significant interaction nor main effect of diet and TRF on these behaviors (Figure 2E,F). No significant correlations were found between body fat mass percentage and memory performances for any of the memory tests, in HFS diet-fed mice, indicating that improvement of memory performances by TRF was independent of fat mass quantity (Supplementary Fig. S1).

**Figure 2:**
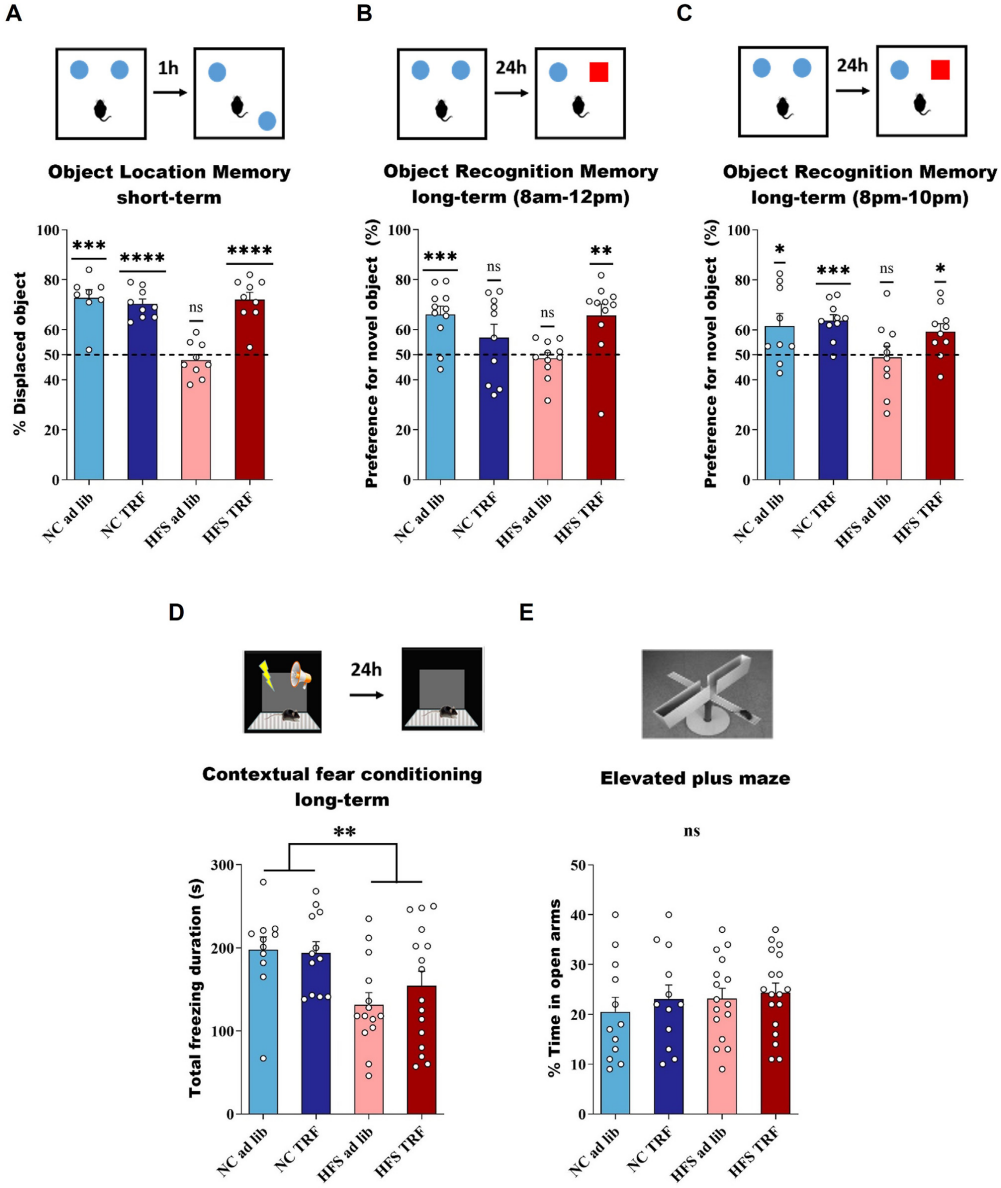
**Juvenile ad lib HFS diet induces memory deficits that are prevented by TRF**. **A**: Object Location Memory test, *n* = 8–9/per group; **B–C**: Object Recognition Memory test in the morning (*n* = 10–11 per group) and the evening, *n* = 9–10 per group. **D**: Contextual Fear Conditioning, *n* = 12–16 per group. **E**: Elevated Plus Maze *n* = 12–18 per group. Behavioral tests were done in the morning (or early afternoon for OLM) unless indicated. For OLM and ORM, each experimental group was assessed for a value of preference, analyzed by a one-sample t test between the group mean and 50%, that is, chance level but also 2-way ANOVA (see main text). CFC and EPM data were analyzed by 2-way ANOVA (Diet and TRF factors). Data are represented as mean ± SEM, ns: *p* > 0.05; ∗*p* < 0.05; ∗∗*p* < 0.01; ∗∗∗*p* < 0.001; ∗∗∗∗*p* < 0.0001).

### 3.3 HFS ad lib diet impacts memory-induced hippocampal translatome that is partially rescued by TRF

In order to get insights into the mechanisms involved in HFS-induced memory impairments and their prevention by TRF, we examined the hippocampal molecular changes that accompanied the behavioral effects using a translatomic approach. Given that only a fraction of cells is activated in the hippocampus during memory acquisition and consolidation, we employed a TRAP technique to immuno-precipitate mRNAs currently being transcribed, using an antibody targeting the terminal phosphorylation sites (Ser244/247) of ribosomal protein S6 (pS6-TRAP) as in [34,35] followed by RNA sequencing to quantify mRNAs. Immunolabeling of pS6^Ser244/247^ was not found significantly different between groups in hippocampus CA1, CA3 and DG subregions (Supplementary Fig. S2). The design of the pS6 TRAP-RNAseq experiments is depicted in Figure 3A. In a first experiment, mice from NC ad lib, HFS ad lib, and HFS TRF groups were each split into two subgroups, one being euthanized under home cage conditions at zt3 (HC zt3) and the other half culled 1 h after a 10-min training of the ORM task that occurred between zt2 and zt4 (ORM+1 h) to study the early phase of memory-induced genes. In a second experiment, mice from NC ad lib, HFS ad lib, and HFS TRF groups, were euthanized either under home cage conditions at zt15 (HC zt15) for half of each group or 12 h (ORM+12 h, around zt14-zt16) after a 10-min training of the ORM task that occurred between zt2 and zt4 for the other half of each group, to test the late phase of memory-induced genes as well as the diurnal expression variation by comparing HC zt15 vs. HC zt3 data. The group NC TRF was not analyzed because both metabolic rhythms and behaviors were similar to the NC ad lib group.

**Figure 3:**
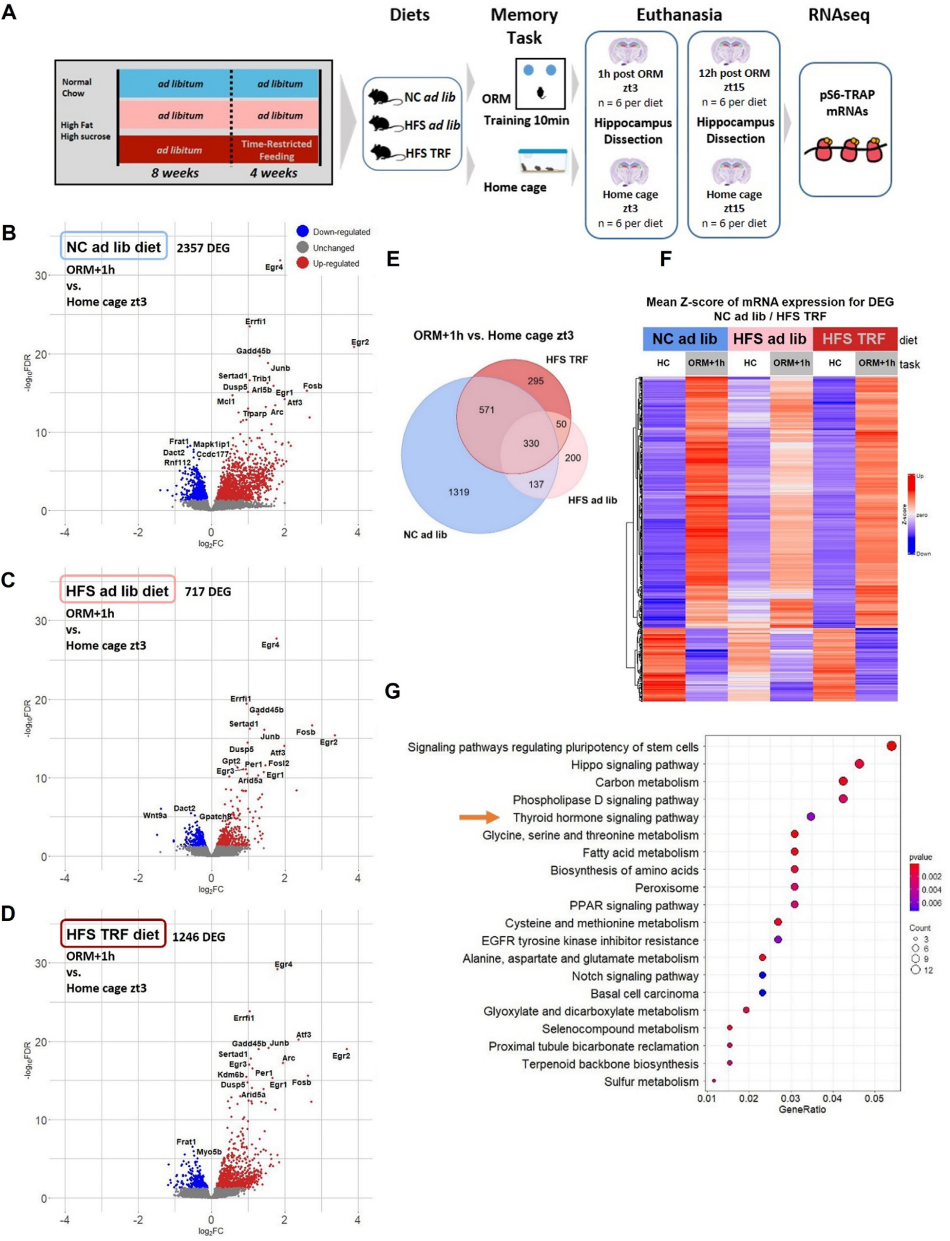
**Juvenile ad lib HFS diet modulates diurnal hippocampal translatome that is partially prevented by TRF**. **A**: Experimental design. **B–D**: Volcano plots of the differential gene expression analyses in each diet comparing the early transcriptional response to memory (ORM+1 h vs. HC zt3). **E**: Venn diagram of the differentially expressed genes (DEG) in each diet (ORM+1 h vs. HC zt3). **F**: heatmap of mean z scores of the DEG that are shared between NC ad lib and HFS TRF groups. **G**: Enrichment analysis of the DEG that are shared between NC ad lib and HFS TRF groups. Differential gene expression analyses were done with DEseq2 package using a FDR *p* < 0.05.

As illustrated in the Volcano plots comparing the conditions ORM+1 h and HC zt3 of each diet (Figure 3B–D), measuring the early phase of experience-induced genes, there were more genes up-regulated than down-regulated in each diet and the lowest number of differentially expressed genes (DEG) was found in the HFS ad lib group. We focused on the 571 DEG being commonly expressed in both NC ad lib and HFS TRF mice, but unchanged in HFS ad lib after ORM acquisition phase (Figure 3E, Supplementary Table S1). This subset of genes is of particular interest since it may be associated with the biological functions that are rescued by TRF to ameliorate HFS-induced memory deficits. Their mRNA levels, expressed as mean z scores, are illustrated in a heatmap (Figure 3F). Among the top-enriched pathways of these 571 DEG (Figure 3G), thyroid hormone signaling caught our attention due to their known involvement in brain development and memory processes [36,37]. The genes representing the thyroid pathway are Atp1a2, Atp1b2, Dio3, Mapk3, Med12, Med16, Notch1, Pik3ca and Picd4.

When we examined the late-phase translational response of gene expression by comparing condition 12 h after the ORM task (ORM+12) to the home cage control at a similar time point (HC zt15), only 239 genes were modified in NC ad lib group compared to 2357 genes at ORM+1 h, 201 genes in HFS ad lib group compared to 717 at ORM +1 h but 1595 genes in HFS TRF mice compared to 1246 genes at ORM+1 h (Supplementary Fig. S3). Thus, modification of gene expression during memory consolidation takes place mostly at an early phase under ad lib NC or HFS diets but is equally distributed under HFS TRF regimen. Only 18 induced genes are shared between NC ad lib and HFS TRF. These genes are related to neuronal plasticity, through neurogenesis or neurite outgrowth. None of these 18 genes were differentially expressed in NC ad lib and HFS TRF, 1 h after ORM. As for the numerous genes modified in the HFS TRF group, 12 h after ORM we performed further analyses that are detailed in supplementary text and Supplementary Fig. S3.

**Figure 4:**
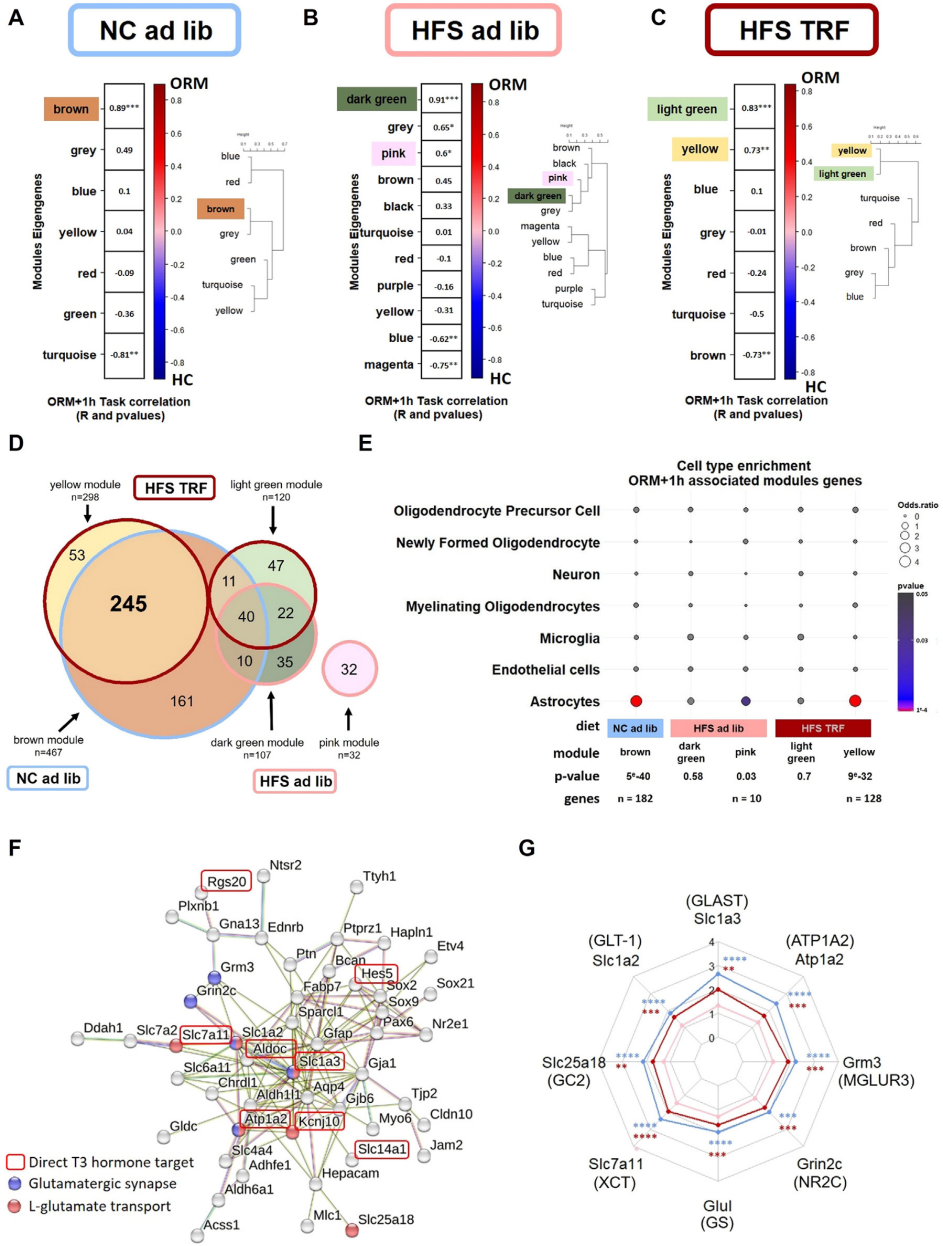
**Co-regulated network analysis highlights astrocytic genes associated with memory**. **A–C**: Modules of co-regulated genes in each diet as defined by WGCNA analyses. Each module is identified with a color name. The color scale on the right indicates the R values for the Pearson correlations (Module-trait correlation between eigengene expression in each module and ORM vs. home cage (HC) condition. P values for Pearson correlation are noted within each module **D**: Venn diagram showing genes shared between modules of co-regulated gene network of each diet **E**: Cell-type analysis of the ORM-correlated genes for each module of each diet. **F**: Major network of the astrocytic genes shared between NC ad lib and HFS TRF modules (STRING software), (See also Fig. S4). **G**: Gene expression (RNAseq data) represented in a radar plot for genes involved in the regulation of glutamate neurotransmission. The stars shown on the top of each gene represent the p adjusted values from the DEseq2 global analysis.

**Fig. S4:**
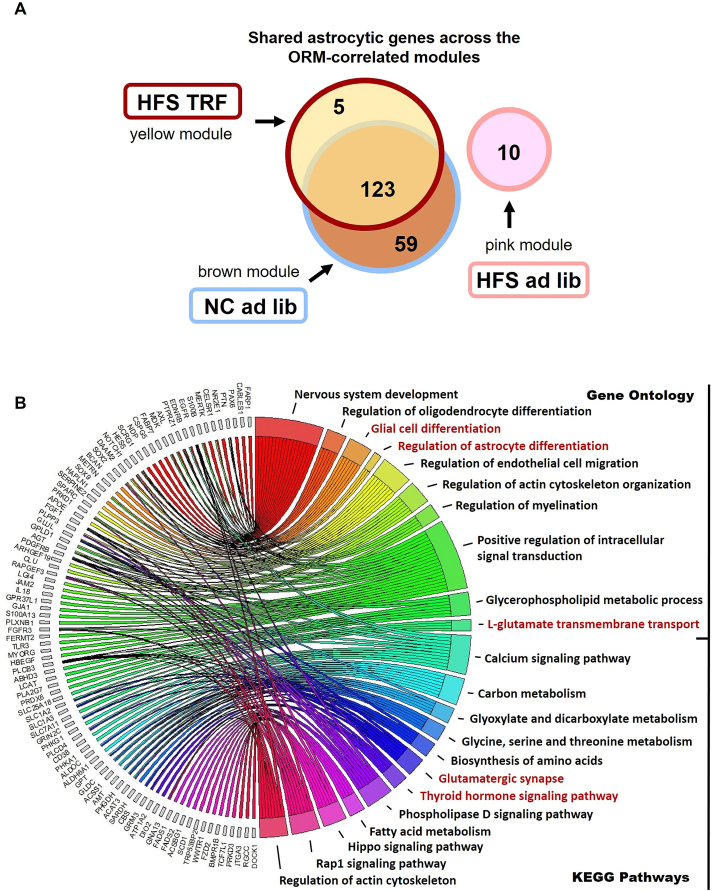
**WGCNA analysis: selection of genes set and enrichment analysis. A**: Venn diagram showing the number of shared genes between the modules defined by the WGCNA analysis of Fig. 4 that are expressed in astrocytes. The 123 astrocytic genes in common between brown and yellow modules of respectively NC ad lib and HFS TRF were further examined in the study. **B**: Enrichment analysis of the 245 genes (not necessarily astrocytic) shared between brown and yellow modules of respectively NC ad lib and HFS TRF.

After identifying the broad patterns of translatome-wide changes associated with diet exposure and memory alterations, we aimed to resolve gene co-expression networks to pinpoint translatome changes that may be critical for understanding how diet and TRF influence early memory consolidation, specifically 1 h after ORM. To achieve this, we employed a weighted gene co-expression network analysis (WGCNA) [29] as a systems biology approach to identify modules (clusters) of highly co-expressed genes from the RNAseq data (ORM+1 h vs HC zt3), for each diet (Figure 4A–C). We then correlated the expression level of each module eigengene (which summarizes the expression of the genes within each module) with the conditions of ORM versus HC. The NC ad lib group exhibited one module (brown), while the HFS ad lib showed two modules (dark green and pink) that were highly correlated with ORM (Figure 4A–B, Supplementary Tables S2–4)). Notably, HFS TRF mice exhibited two modules correlated with ORM, one (yellow module) was significantly enriched with genes expressed in the brown cluster of NC ad lib mice (245 genes/298 total, 82%, Supplementary Table S2) and the other (light green) was enriched with genes from the most significant module of the HFS ad lib group (62 genes/121 total, 51%; Figure 4D). Enrichment analyses of the 245 genes shared between the NC and HFS TRF (Brown-Yellow) modules are presented in Supplementary Fig. S4. In addition to the pathways already noted in the enrichment analysis of Figure 3G, such as thyroid hormone signaling, we observed enrichment in pathways related to glial cell differentiation (including astrocytes and oligodendrocytes), l-glutamate transmembrane transport and the glutamatergic synapse.

Next, a cell type enrichment analysis, performed as described in [31,32], revealed that in mice under the NC ad lib and HFS TRF diets, genes correlating with ORM where predominantly expressed in astrocytes. This was not the case for mice on the HFS ad lib diet, where only 10 genes from the pink modules show significant expression in astrocytes (Figure 4E). We identified 123 astrocyte-expressed genes that were common between the NC ad lib and HFS TRF mice (Supplementary Fig. S4, Supplementary Table S5), which were then subjected to gene network analysis. The major cluster included 49 astrocytic genes, several of which are involved in the regulation of glutamate uptake and transmission (Figure 4F). These genes, specifically GLAST (Slc1a3), GLT1 (Slc1a2), GC2 (Slc25a18), XCT (Slc7a11), Atp1a2, GS (Glul), NR2C (Grin2c) and MGLUR3 (Grm3), were not significantly upregulated in the HFS ad lib group (or show lesser upregulation for XCT), suggesting a deficiency in glutamate reuptake and recycling for mice under this diet (Figure 4G).

Thanks to a recently published mouse brain atlas of thyroid hormones’ direct target genes in astrocytes [38], constructed from recent RNAseq and ChIP-seq databases), we found that 22.4% (55/245 total) of the ORM-correlated genes are targets of thyroid hormones (highlighted in Supplementary Table S5). Some of these thyroid-responsive genes are emphasized in the cluster shown in Figure 4F.

Collectively, the RNAseq analysis on memory-induced genes indicated that the hippocampal translatome is highly modified under HFS ad lib diet at the early phase of gene transcription. Part of the hippocampal translatome is rescued by TRF and includes thyroid hormone pathway as well as astrocytic genes involved in the regulation of glutamate neurotransmission.

### 3.4 HFS ad lib diet impacts day–night variations of hippocampal translatome that is partially rescued by TRF

Since HFS ad lib diet is known to disturb molecular rhythms and TRF to reinstate them, we evaluated day–night gene expression by the comparison of DEG between HC zt3 and HC zt15 (Figure 5A–F) in each diet.

**Figure 5:**
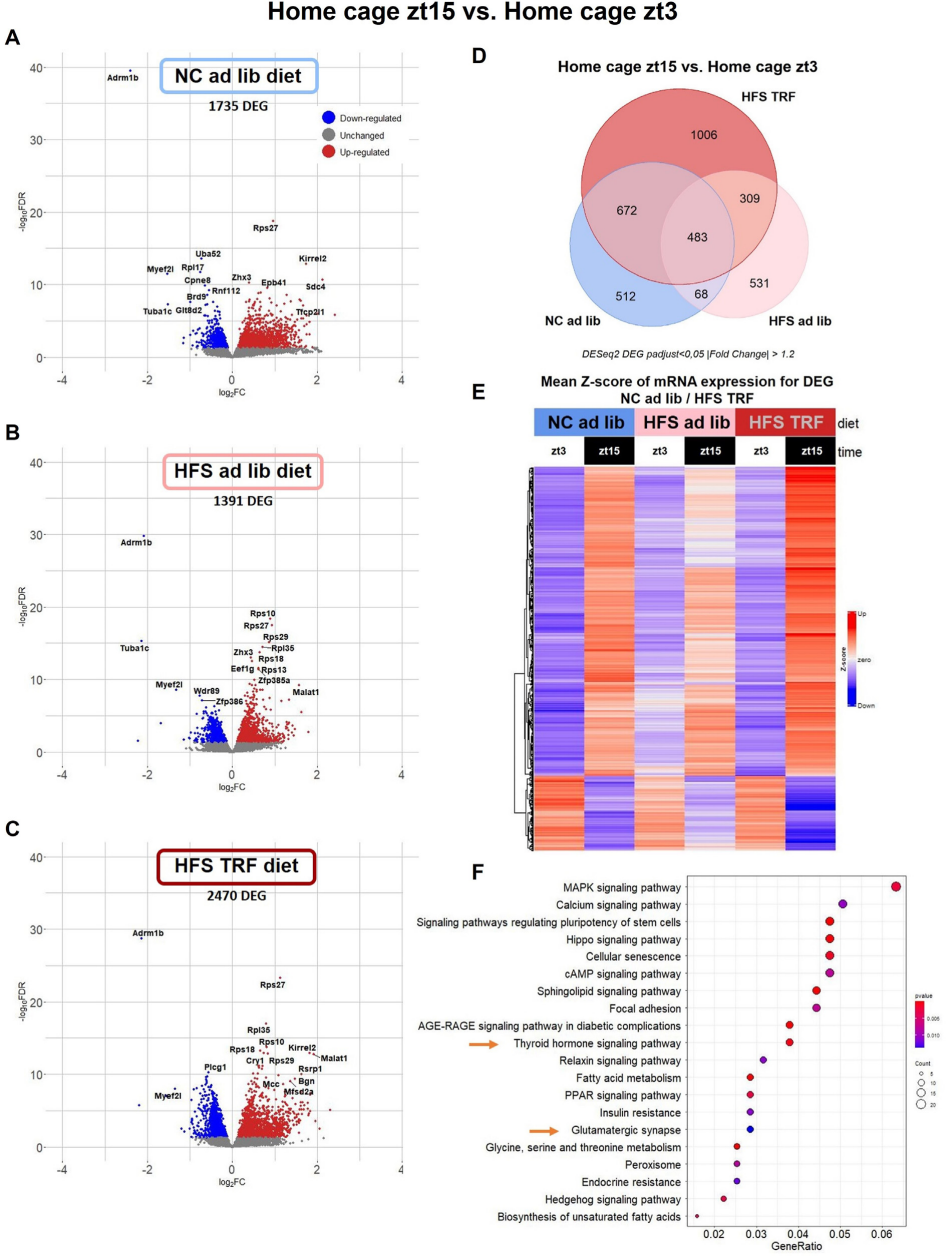
**Juvenile HFS ad lib diet impacts day–night variations of hippocampal translatome that is partially rescued by TRF. A–C**: Volcano plots of the differential gene expression analyses in each diet comparing diurnal variation of expression in home cage condition (HC zt15 vs. HC zt3). **D**: Venn diagram of the differentially expressed genes (DEG) in each diet (HC zt15 vs. HC zt3). **E**: heatmap of mean z scores of mRNA expression of the DEG that are shared between NC ad lib and HFS TRF groups. Differential gene expression analyses were done with DEseq2 package using a FDR *p* < 0.05 **F**: Enrichment analysis of the DEG that are shared between NC ad lib and HFS TRF groups.

A higher number of DEG was found in HFS TRF mice (2470) followed by NC ad lib (1735) and HFS ad lib (1391) as shown on the volcano plots and the Venn diagram (Figure 5A–D). Importantly, a high number of genes (672) lost their day–night oscillation in HFS ad lib mice while this diurnal variation was rescued by TRF (Figure 5D, Table S6), as illustrated on the heatmap of mean z score of mRNA expression (Figure 5E). Interestingly, 19% (130/672) of these genes were part of the brown-yellow gene modules correlated with ORM. Remarkably, enrichment analysis revealed that this subset of genes was enriched in thyroid hormone signaling and glutamatergic synapse (Figure 5F) pathways already identified above, hence indicating that rhythmicity of such pathways are of particular relevance for the regulation of memory processing. Lastly, the core clock genes Per3, Npas2 and Nr1d2 (REVERβ) showed a loss of day–night expression variation in HFS ad lib but not in the two other groups, further indicating the perturbation of hippocampal circadian rhythms in mice fed HFS ad lib (Supplementary Fig. S5).

Overall, these results demonstrate that a large fraction of genes associated with memory function, and perturbed in HFS ad lib mice, exhibit baseline time-of day-specific expression, and indicate a central role of thyroid hormones signaling and glutamatergic synapse. These data suggest that circadian dysfunction may impact the hippocampal translatome of HFS ad lib mice that is reinstated by TRF.

### 3.5 Thyroid hormone signaling pathway is altered by HFS and partially rescued by TRF

Based on the aforementioned results, we decided to focus on thyroid hormone signaling pathway and to validate our translatomic analysis. First, we identified that Dio3, encoding iodothyronine deiodinase 3 enzyme, which metabolizes the active thyroid hormone T3 in inactive reverse T3 (rT3), was the most down-regulated gene, 1 h after ORM, in NC ad lib and HFS TRF mice while its expression was unchanged in HFS ad lib mice. Dio2, encoding for iodothyronine deiodinase 2 enzyme, which converts T4 to active T3, is highly induced by the memory task in all groups, although less significantly in HFS ad lib group (Supplementary Fig. S6). Consequently, the ratio of Dio2/Dio3 hippocampal expression revealed a decreased ratio in HFS ad lib mice, suggesting a reduced T3 availability within the hippocampus of these mice, that is rescued by TRF (Figure 6A). To further document this result we measured thyroid hormones within hippocampus of each mice group by mass spectrometry. T3 and T4 were detected at significant levels whereas no rT3 was detectable in the hippocampus of any of the mice. Although not reaching statistical levels by 2-way ANOVA (interaction *p* = 0.10) a clear trend for reduced T3/T4 levels was found in HFS ad lib group compared to NC controls (*p* = 0.068) and to HFS TRF (*p* = 0.055), comforting the idea of local hypothyroidism (Figure 6B). The reduced T3/T4 ratio levels in HFS TRF mice is due to higher T4 levels, suggesting impaired Dio2 functioning in this group (Supplementary Fig. S7). The thyroid transporter MCT8 (Slc16a2) gene expression was found unchanged in all diet. The other thyroid transporter OATP1 (Slco1c1) was up-regulated after ORM training in NC ad lib group only (Supplementary Fig. S6). Thyroid hormones have two receptors THRα and THRβ, each of them displaying two isoforms. Thrb2 but not Thrb1 isoform expression was detected in hippocampus by the RNAseq analysis, and was found unchanged after ORM in all diet (Fig. S6). As for THRα, both Thra1 and Thra2 were highly expressed in the hippocampus. Thra1 expression was not altered by ORM or diet while the expression of Thra2 was significantly down-regulated by ORM in NC ad lib and HFS TRF mice (Fig. S6). The Thra1/Thra2 ratio increased after ORM in NC ad lib and HFS TRF but not in HFS ad lib (Figure 6C). Because THRα1 and THRα2 isoforms are generated by the differential splicing of the Thra gene, we examined differential exons usage on the RNAseq data of each diet. The DEXseq analysis revealed that a THRα1-specific exon is differentially used in mice under NC ad lib (*p* = 0.037) and HFS TRF (*p* = 0.030) after ORM but not in mice under HFS ad lib diet (Figure 6D). This result is in agreement with the reduced ratio of Thra1/Thra2 expression and a putative lower THRα1 signaling in HFS ad lib mice.

**Figure 6:**
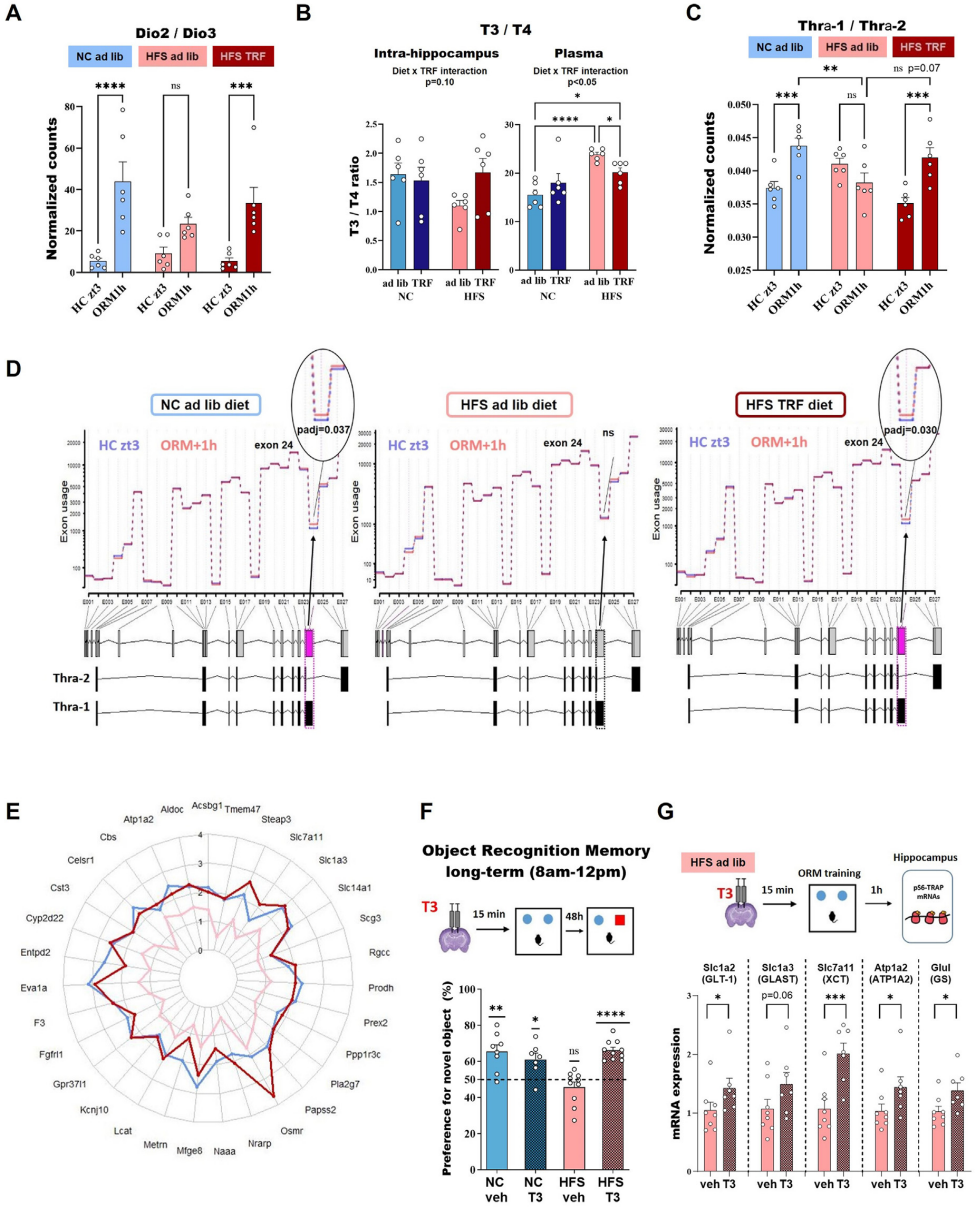
**Thyroid hormone signaling pathway contributes to HFS diet-induced memory impairments and TRF prevention**. **A**: Gene expression ratio of Dio/Dio3 (normalized counts from DEseq2 analysis, see also Fig. S6). **B**: T3/T4 hormone levels within hippocampus measured by mass spectrometry (see also Fig. S6). **C**: Ratio of Thra1/Thra2 (normalized counts from the Deseq2 analysis, see also Fig. S6). **D**: DEXseq analysis for Thra gene in each diet. The differentially used exon is highlighted in pink and the p adjusted value from the DEXseq analysis indicated for each diet. **E**: Radar plot of genes correlated to ORM (from Figure 4), targets of thyroid hormones in astrocytes that display loss of day–night variation of expression (HC zt15 vs. HC zt3 comparison) in HFS ad lib mice (pink line) but significantly vary in NC ad lib (blue line) and HFS TRF (dark red line) groups. **F**: Long-term Object Recognition Memory (ORM) results of NC ad lib and HFS ad lib mice infused with either vehicle or T3 within hippocampus, *n* = 7–10 mice per group, each experimental group was assessed for a value of preference, analyzed by a one-sample t test between the group mean and 50%, that is chance level, as well as 2-way ANOVA (Diet x T3 treatment, see main text).). **G**: Relative gene expression levels of astrocytic genes in HFS ad lib mice following vehicle or T3 infusion in hippocampus, measured by qPCR 1 h after ORM training on pS6 TRAP mRNA, *n* = 6 mice per group, statistical significance was determined using one-tailed t-test. Data are represented as mean ± SEM, ns: *p* > 0.05; ∗*p* ≤ 0.05; ∗∗*p* < 0.01; ∗∗∗*p* < 0.001; ∗∗∗∗*p* < 0.0001).

Among the ORM-correlated genes rescued by TRF (brown and yellow modules overlap of Figure 4), we mentioned above that 55 of them (22,4%) were direct target of thyroid hormones. Looking at their expression at zt15 vs. zt3 in home cage condition revealed that 32 out of these 55 genes lost their diurnal expression variation under HFS ad lib diet but recovered it under TRF (Figure 6E). Thus, thyroid hormones regulated genes in astrocytes may contribute to memory formation and their diurnal variation may be the mechanism by which TRF ameliorates memory.

### 3.6 Thyroid hormone infusion within the hippocampus rescues memory in HFS ad lib mice

Given the convergent lines of evidence pointing towards the involvement of local hypothyroidism in the impaired memory of HFS ad lib mice, we went on by assessing the functional impact of thyroid hormone on hippocampal-dependent memory processes in NC ad lib and HFS ad lib groups. For this, T3 hormone was locally and bilaterally infused within the dorsal hippocampus (1.2ng/0.3μl/side) 15 min before ORM training. This hippocampal T3 infusion rescued long-term ORM deficit in HFS ad lib mice without affecting performance of NC ad lib mice (one-sample t test against 50%: *p* < 0.02 for all groups except HFS ad lib; 2-way ANOVA interaction diet x TRF *p* < 0.001, HFS ad lib *p* < 0.01 compared to all other groups, Figure 6F) demonstrating that deficient thyroid hormones action in hippocampus plays an important role in the memory impairments of HFS ad lib mice. Finally, we assessed astrocytic gene expression levels 1 h after ORM training in HFS ad lib fed mice. T3 hormone up-regulated significantly the astrocytic gene expression of the 5 genes tested (Figure 6G).

## 4 Discussion

TRF without calorie restriction is currently the preferred intermittent fasting strategy for combating obesity and its associated comorbidities because of its simplicity of implementation in human settings and the beneficial effects observed in both animal models and humans. Research examining the impact of TRF on brain health and disease is just beginning to emerge [22,39,40]. This study contributes to that body of work by demonstrating that a 4-week TRF protocol positively affects both short and long-term memories in mice fed HFS diet ad lib during peri-adolescence. We provide evidence that thyroid hormone signaling plays a significant role in the memory impairments caused by the ad lib HFS diet and their rescue by TRF, via the modulation of astrocytic genes involved in the regulation of glutamate transmission.

First, we observed that mice under a 12-week ad lib HFS diet tend to eat more food during the inactive light phase (zt0-zt12) compared to those on NC diet, as previously noted by others [11,12,41,42]. Under TRF, HFS-fed mice still exhibit higher calorie intake than NC mice; however, their feeding patterns aligned more closely with day/night cycles. Increased energy expenditure likely accounts for the reduced fat mass gain in HFS TRF mice compared to those on ad lib HFS diet, as locomotor activity remained unchanged and total food intake was not reduced by TRF. While similar effects of TRF on metabolism, particularly regarding energy expenditure, have been documented in other studies [20,41], results have not always been consistent [39,43]. These discrepancies may arise from variations in diet composition, duration and age of exposure to the HFS diet, and differences in the scheduling and length of TRF across studies.

In addition to its beneficial metabolic effects, we found that TRF prevented both short-term OLM and long-term ORM deficits in our model of juvenile HFS diet consumption. Notably, the alteration in contextual fear conditioning was not rescued by TRF, possibly due to the complexity of the memory task. Other studies have also reported positive effects of TRF on short- and long-term memory [22,39]. Our data suggest that TRF’s direct action on the brain is more likely responsible for the observed memory improvements, as we found no correlation between body fat percentage and memory performance in our animal cohorts.

The formation of long-term memory is dependent on the activation of gene transcription and *de novo* protein synthesis [44]. Regarding the early phase of transcriptional response (ORM+1 h vs. HC zt3), our RNAseq data confirmed, for each diet and regimen, the up-regulation of activity dependent transcription factors such as Egr2, Fosb, Fos, Egr1, Arc, Nr4a1 and DNA methylation modifiers such as Gadd45b, which are crucial for neuronal activity during memory formation [45]. When we examined the hippocampal translatome 12 h after ORM, we found a significantly lower number of induced genes in the NC and HFS ad lib groups compared to 1 h post ORM, indicating that transcriptional programs involved in memory consolidation take place at an early stage. In contrast, mice on the HFS TRF diet maintained a high number of induced genes 12 h after ORM, although different from those induced 1 h post ORM. Further time points would be necessary to explore dynamic changes in gene expression over time in each dietary group.

Enrichment analyses of the set of genes rescued by TRF highlighted the role of thyroid hormone signaling, which is noteworthy given that these hormones are critical for hippocampus-dependent cognitive functions such as learning and memory [26,46]. We further investigated the RNAseq data of the early phase of transcription (ORM+1 h vs. HC zt3) to examine co-regulated gene networks, their correlation with memory formation and the involved cell types. These analyses indicated that co-regulated gene networks associated with memory formation in NC ad lib and HFS TRF mice were predominantly expressed in astrocytes where they play essential role in regulating central nervous system development, synaptic plasticity and glutamate buffering. Notably, several astrocytic glutamate transporters, such as GLAST and GLT-1 exhibited significant up-regulation in both NC ad lib and HFS TRF mice but remained unchanged in HFS ad lib mice hour after ORM. These transporters are crucial for glutamate clearance, ensuring the reuptake of glutamate from the synaptic cleft. The network also included Atp1a2 and Atp1b2 genes, which encode subunits of Na^+^, K^+^-ATPase pumps, located in the astrocyte membrane and necessary for the activity of glutamate transporters [47]. The gene Glul, which encodes glutamine synthase (GS), also appeared in the memory-associated network and is responsible for terminating glutamate transmission by converting glutamate into glutamine within astrocytes. Furthermore, the metabotropic glutamate receptor MGLUR3 and the ionotropic glutamate receptor NR2C, encoding the genes Grm3 and Grin2c respectively, displayed similar mRNA expression patterns to the glutamate transporters. The lack of up-regulation of these genes in HFS ad lib mice post-ORM likely lead to altered glutamate clearance, which could affect tonic excitation and synaptic currents mediated by glutamate receptors [48]. Fascinatedly, GLAST and GLT-1 were found to be down-regulated in the ventral hippocampus of HFS fed mice [49] and GLT-1 function was impaired in the orbitofrontal cortex of HFS fed rats [50]. Also of great interest for our study, thyroid hormones have been reported to regulate the pattern of astrocytes maturation in cerebellum via THRα1 [51] and to up-regulate GLAST and GLT-1 to protect astrocytes and neurons from glutamate toxicity [52]. A recent mouse brain atlas of thyroid hormone target genes confirmed direct regulation of astrocytic genes by thyroid hormone [38], revealing that over 22% of the genes associated with ORM in our study are target of thyroid hormones, including GLAST, XCT, ATP1A2 and GS. Utilizing RNASeq data obtained at two time points 12 h apart (HC zt15 vs HC Zt3), we identified genes that loss day–night expression variation in HFS ad lib mice but were restored by TRF. A significant proportion of these genes belongs to the previously identified memory-associated network, and is enriched in pathways related to thyroid hormones signaling and glutamatergic synapses.

Given this information, we decided to focus on thyroid hormone signaling and its potential role in the memory deficits observed in HFS ad lib mice and their restoration in HFS TRF mice. Differential gene expression analysis revealed that Dio3 was the most down-regulated gene post ORM in both NC ad lib and HFS TRF mice in the hippocampus. This prompted us to compare the Dio2/Dio3 mRNA ratio levels across diets, a measure considered a reliable proxy for estimating local bioavailability of the active form of thyroid hormone, T3. The Dio2/Dio3 ratio significantly increased 1 h post-ORM in NC ad lib and HFS TRF but not in HFS ad lib, suggesting a potential deficit of active T3 in the hippocampus of HFS ad lib mice. Although hippocampal T3 levels were not different between groups, T4 levels were slightly elevated in HFS ad lib mice, leading to a trend of reduced T3/T4 ratio, which was restored following TRF intervention. These changes were not observed in plasma, indicating a local hippocampal regulation of thyroid hormones. Regarding THRα, the ratio of the two isoforms suggested that THRα1 signaling might be increased after ORM in both NC ad lib and HFS TRF, potentially due to reduced inhibition by THRα2, which did not occur in HFS ad lib mice. This finding was supported by the intriguing observation of differential exon usage favoring THRα1 after ORM in NC ad lib and HFS TRF groups but not in HFS ad lib mice. Whether the elevated levels of dominant-negative THRα2 isoform after ORM affect THRα1 signaling during memory formation in the hippocampus of HFS ad lib mice remains an intriguing possibility that warrants further investigation. Interestingly, a recent report described a patient with a specific genetic mutation in the THRα gene sequence, resulting in an increased THRα2 antagonism, which led to neuronal hypothyroidism and intellectual disability [53].

Finally, we demonstrated the significance of deficient T3 action in the hippocampus of HFS ad lib mice, by showing that direct infusion of T3 into the hippocampus restored long-term ORM of these mice and induced expression of astrocytic genes involved in glutamate reuptake and recycling. This finding aligns with the reduced gene expression of Dio2/Dio3 and lower T3/T4 levels observed in hippocampus of HFS ad lib mice, supporting the hypothesis of local hypothyroidism.

A limitation of this study is that we did not analyze females, whose metabolic response to HFS diet differ from those of males. Female mice do not exhibit impairments in contextual fear memory but show alterations in long-term ORM [24]. Future research should investigate the beneficial effects of TRF and the role of thyroid hormones in female mice fed HFS diets from weaning onward.

In conclusion, this study demonstrates that male mice fed a HFS diet since weaning present deficiency in hippocampal T3 hormone action and strongly suggest that THRα1 signaling is impaired, leading to long-term memory impairments, through dysregulation of astrocytic genes implicated in glutamate clearance. TRF restores a higher day–night expression variation of part of these dysregulated genes modulated by thyroid hormones, that may prevent the long-term HFS diet induced memory deficits. Time-restricted eating has proven to be efficient to ameliorate metabolic parameters in obese patients including adolescents [54,55]. Now, it will be interesting to measure the effects of time-restricting eating on memory performances in adolescent patients with obesity.

## CRediT authorship contribution statement

**Jean-Christophe Helbling:** Methodology, Investigation, Formal analysis, Data curation. **Rachel Ginieis:** Writing – review & editing, Methodology, Investigation, Formal analysis. **Pierre Mortessagne:** Investigation. **Mariano Ruiz-Gayo:** Writing – review & editing, Methodology, Investigation, Formal analysis, Conceptualization. **Ioannis Bakoyiannis:** Writing – review & editing, Investigation. **Eva-Gunnel Ducourneau:** Resources, Methodology. **Dominique Ciocca:** Investigation. **Illona-Marie Bouleté:** Investigation. **Alexandre Favereaux:** Resources, Methodology, Investigation. **Aurélia Ces:** Investigation. **Enrica Montalban:** Writing – review & editing, Methodology. **Lucile Capuron:** Writing – review & editing. **Freddy Jeanneteau:** Writing – review & editing, Funding acquisition. **Guillaume Ferreira:** Writing – review & editing, Resources, Methodology, Funding acquisition, Formal analysis, Conceptualization. **Etienne Challet:** Writing – review & editing, Resources, Methodology, Investigation, Funding acquisition, Formal analysis, Data curation, Conceptualization. **Marie-Pierre Moisan:** Writing – review & editing, Writing – original draft, Visualization, Validation, Supervision, Project administration, Methodology, Investigation, Funding acquisition, Formal analysis, Data curation, Conceptualization.

## Declaration of competing interest

The authors declare that they have no known competing financial interests or personal relationships that could have appeared to influence the work reported in this paper.

## Figures and Tables

**figs1:**
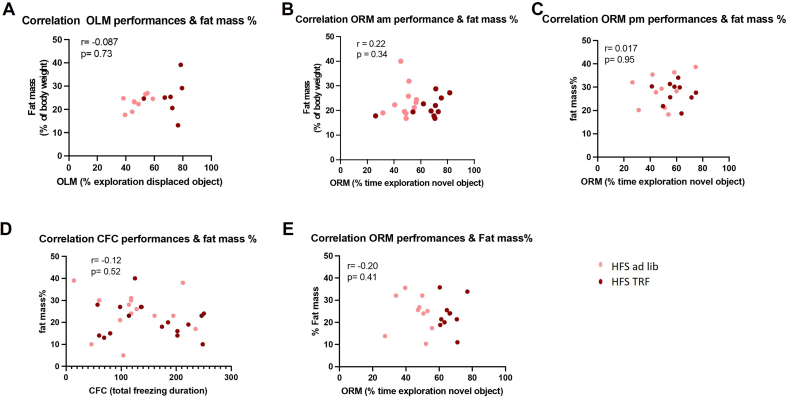
**Fig. S1: Correlation analyses between memory performances and fat mass percentage.** Fat mass was measured by EchoMRI. Different animal cohorts were used for each behavioral test and time. The corresponding figure of the main text showing memory performances is indicated under each graph.

**figs2:**
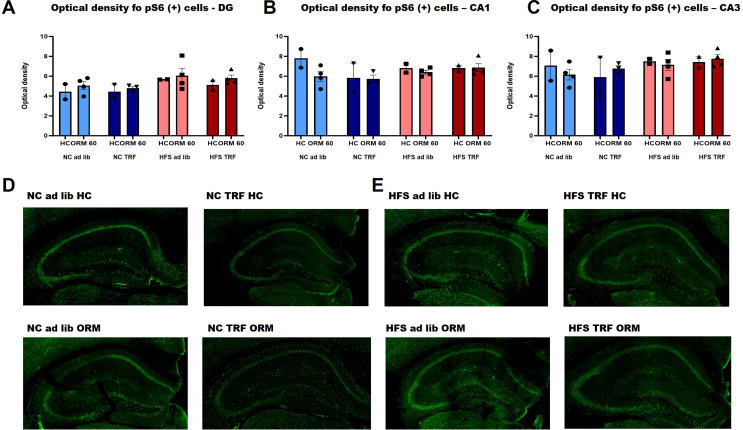
**Fig. S2: Immunofluorescence labeling of pS6-Ser**^**244/247**^**on hippocampal slices.** Data were analyzed by non-parametric *t*-test for each hippocampal subregions, CA1 (**A**), CA3 (**B**) and dentate gyrus, DG (**C**). Representative pictures of labeled slices from each diet are shown for NC **(D)** and HFS **(E)** diets.

**figs3:**
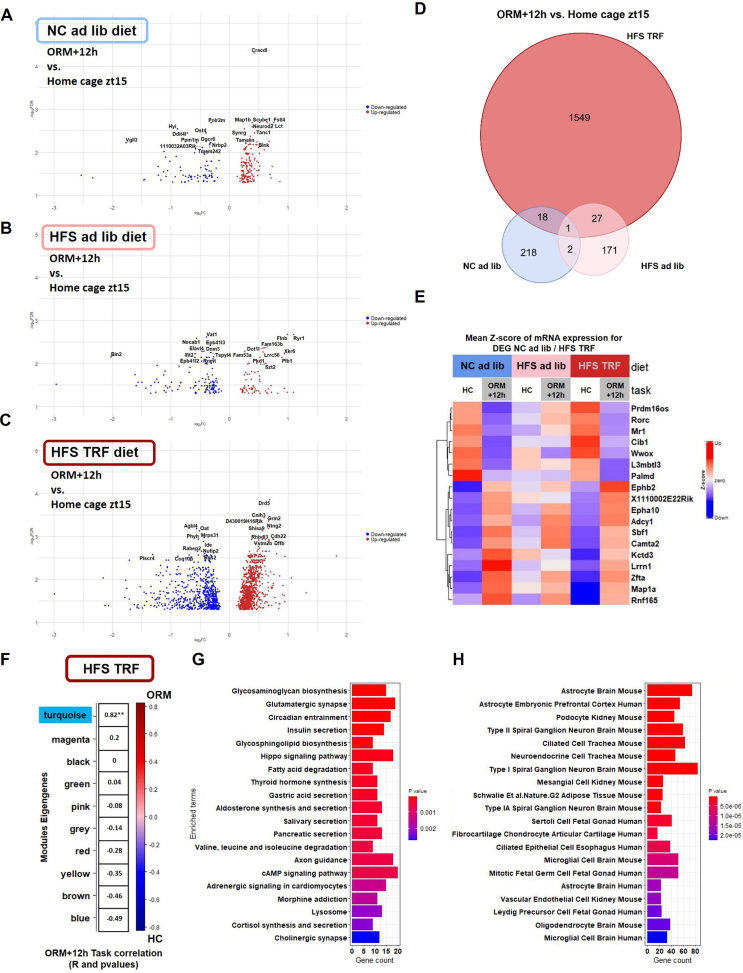
**Fig. S3: HFS diet moderately alters hippocampal translatome 12h after ORM training**. **A-C**: Volcano plots and **D**: Venn diagram of DEG comparing ORM+12 vs zt15 groups. **E**: Heatmap of gene expression from DEG shared between NC and HFS TRF groups. **F:** WGCNA analyses in the group HFS TRF. **G:** Gene enrichment analysis from the turquoise module. **H:** Cell type enrichment analysis from the turquoise module.

**figs5:**
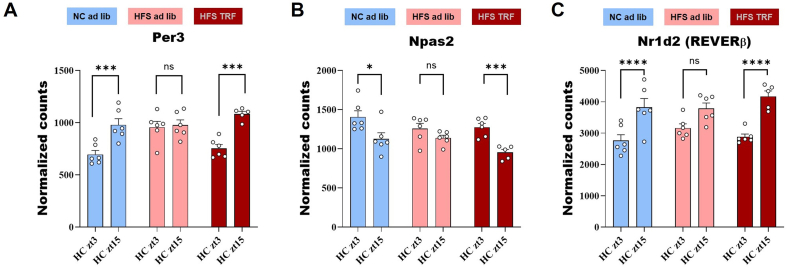
**Fig. S5: Hippocampal gene expression of core clock genes.** Gene expression of the core clock gene Per3, Npas2 and Nr1d2 from the HC zt15 vs HC zt3 comparison. Data were analyzed by DEseq2 package. Data are represented as mean ± SEM, ns: *p* > 0.05; ∗*p* < 0.05; ∗∗*p* < 0.01; ∗∗∗*p* < 0.001; ∗∗∗∗*p* < 0.0001).

**figs6:**
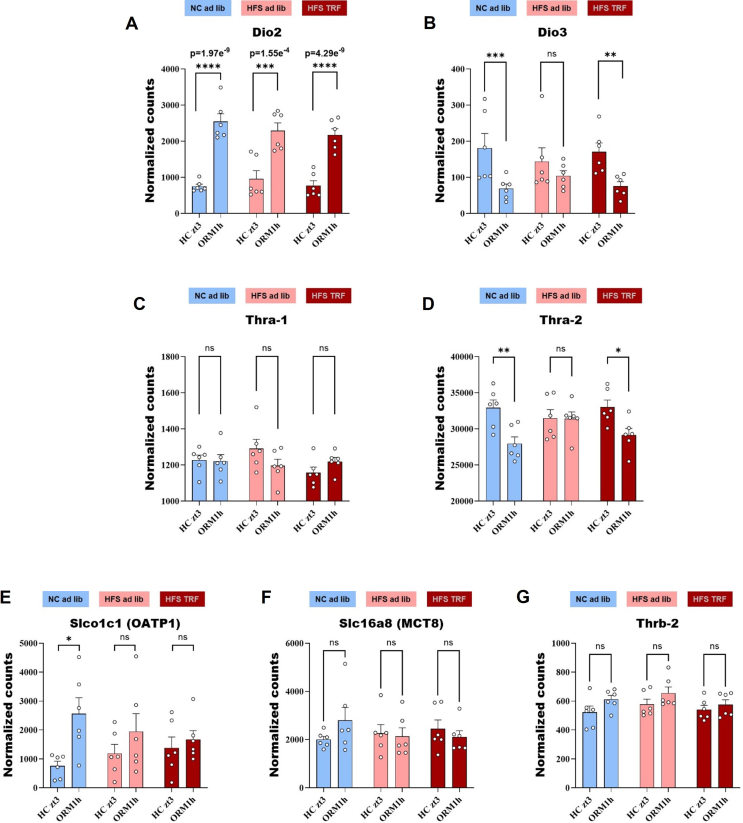
**Fig. S6: Hippocampal gene expression of thyroid hormone signaling genes. A-G:** Gene expression from the ORM+1 h vs. HC zt3 comparison. Data were analyzed by DEseq2 package for each diet. Data are represented as mean ± SEM, ns: *p* > 0.05; ∗*p* < 0.05; ∗∗*p* < 0.01; ∗∗∗*p* < 0.001; ∗∗∗∗*p* < 0.0001).

**figs7:**
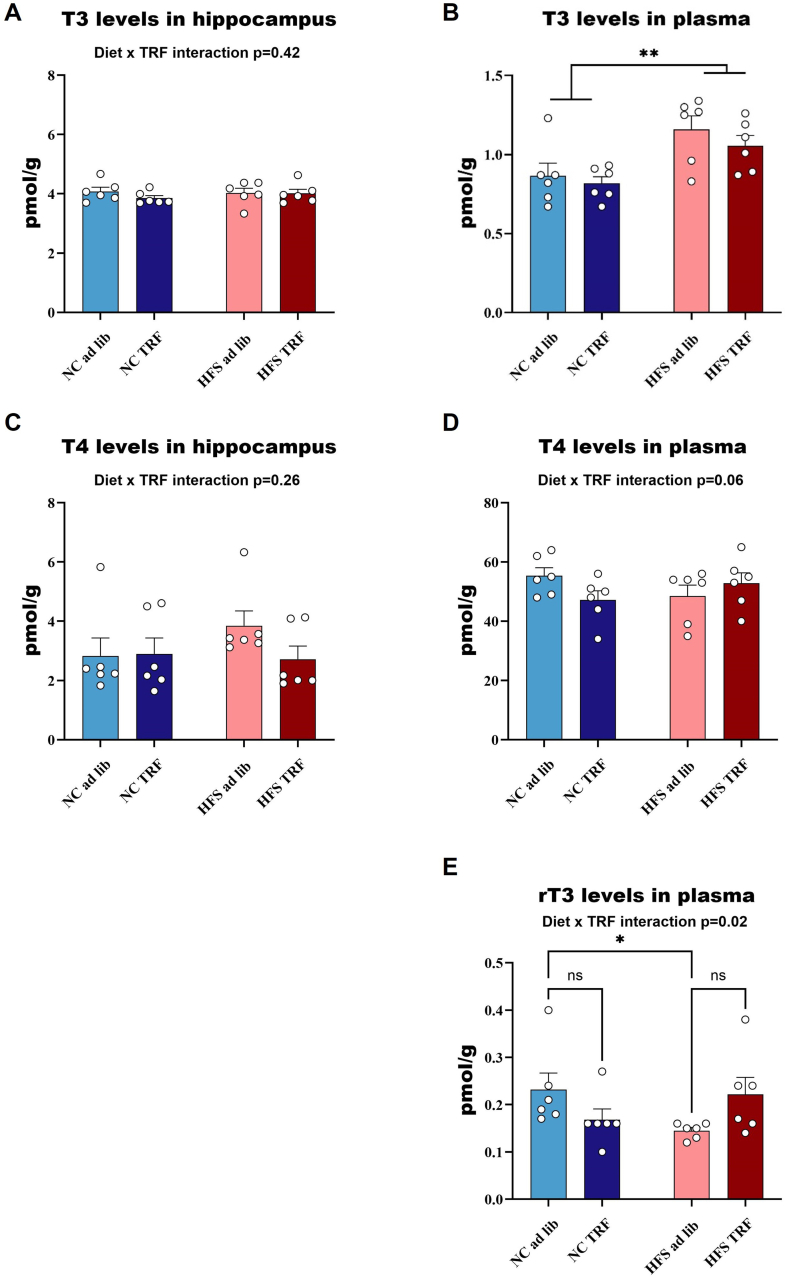
**Fig. S7: Hippocampal and plasma thyroid hormones levels.** Thyroid levels measured by mass spectrometry in hippocampus (**A**,**C)**; in plasma **(B,D,E)**. Data were analyzed by 2-way ANOVA (Diet and TRF factors). Data are represented as mean ± SEM, ns: *p* > 0.05; ∗*p* < 0.05; ∗∗*p* < 0.01.

## Data Availability

Data will be made available on request.
