# Supplementary material for: Time-restricted feeding prevents memory impairments induced by obesogenic diet consumption, via hippocampal thyroid hormone signaling
Source: Mol Metab. 2024 Nov 6;90:102061. doi: 10.1016/j.molmet.2024.102061 (PMC11609524; doi:10.1016/j.molmet.2024.102061)
Supplement: Multimedia component 1 [file mmc1.docx]

**Time-restricted feeding prevents memory impairments induced by obesogenic diet consumption, via hippocampal thyroid hormone signaling**

Jean-Christophe Helbling et al.

**SUPPLEMENTARY TEXT IN RESULTS**

**Juvenile ad lib HFS diet induces, and TRF prevents, alteration of circadian metabolic parameters**

Detailed data and statistics for each metabolic parameter:

**Food intake:**

We found higher mean calorie intake in HFS than NC groups as expected (main effect of diet on food intake mesor F_(1, 36)_ = 12.30; p=0.0012), higher food intake amplitude in HFS TRF compared to both HFS ad lib and NC TRF (diet x TRF interaction on food intake amplitude F_(1, 36)_ = 6.171, p=0.0178) and an earlier food intake acrophase in ad lib than TRF animals (main effect of TRF on food intake acrophase, F_(1, 36)_ = 4.390; p=0.0433)(Fig. 1E-F). From the food intake measurements, we were able to calculate the diurnal percentage of food intake which was 33 ± 5% in HFS ad lib mice versus 21 ± 2% in NC *ad lib* mice (p=0.06, unpaired t test) (Fig.1C). Total 24h calorie intake was significantly impacted by HFS but not by TRF, (2-way ANOVA, main diet effect F_(1, 36)_ =11.45; p=0.0017) (Fig. 1D).

**Respiratory Exchange Ratio (RER)**

For RER, whose decreased and increased values reflect, respectively, higher utilization of lipids and carbohydrates, hourly mean RER was significantly lower in HFS ad lib than both NC ad lib (p<0.001) and HFS TRF (p=0.042) and there was no differences between HFS TRF and NC TRF (p=0.71) (diet x TRF interaction on mesor, F_(1,36)_= 5.905, p = 0.0202). Then, the acrophase was shown to be earlier in HFS than NC, and the amplitude was lower in HFS than NC groups but no effect of TRF was detected (main effects of diet on RER acrophase (F_(1, 36)_ = 54.55; p<0.0001 and RER amplitude, F_(1, 36)_ = 4.680; p=0.0372) (Fig.1G-H).

**Energy Expenditure (EE)**

As for EE, we found higher levels of mean EE in HFS TRF mice than both HFS ad lib mice (p<0.0001) and NC TRF mice (p<0.0001) (diet x TRF interaction mesor, F_(1, 36)_= 78.14, p<0.0001). When looking at the EE amplitude, NC mice showed a higher amplitude than their HFS counterparts, and TRF animals had a higher EE amplitude than ad lib mice (main effects of diet, F_(1, 36)_= 9.723, P=0.0036 and TRF, F_(1, 36)_= 26.49, P<0.0001). There was no significant interaction nor main effects of diet and TRF on EE acrophase (Fig.1I-J).

**Locomotor activity**

Finally, there was no significant interaction nor main effect of diet and TRF on locomotor activity mesor, amplitude or acrophase (Fig.1K-L).

In sum, TRF on HFS diet, by resynchronizing food intake to day/night cycles, restored food intake amplitude, mean RER, mean EE but had no effect on locomotor activity.

**HFS ad lib diet impacts memory-induced hippocampal translatome that is partially rescued by TRF**

***Additional results of pS6 TRAP hippocampal translatome at ORM+12 hours regarding HFS TRF mice***

For HFS TRF mice, 1595 genes were differentially regulated between ORM+12 hour and home cage controls. We found that 105 genes (so 6.5% of the total) were modulated at both ORM+1 hour and ORM +12 hours time points. However, when these genes were induced at 1 hour post ORM they were down-regulated 12 hour later and vice-versa (supplementary Table S8). Then, we employed WGCNA (see method and results about ORM+1 hour analyses) to identify whether part of the differentially expressed genes were correlated with ORM condition. We detected one such module (turquoise), which contained 838 genes related to various pathways (supplementary Table S8), including the glutamatergic synapse, circadian entrainment and thyroid hormone synthesis. Cell-type enrichment data highlighted again astrocytes as the main cell type associated with memory in this HFS TRF group (supplementary Fig.S3).
